# Assessing the effects of limestone dust and lead pollution on the ecophysiology of some selected urban tree species

**DOI:** 10.3389/fpls.2023.1144145

**Published:** 2023-05-15

**Authors:** Muhammad Azeem Sabir, Wei Guo, Muhammad Farrakh Nawaz, Ghulam Yasin, Muhammad Talha Bin Yousaf, Sadaf Gul, Tanveer Hussain, Shafeeq Ur Rahman

**Affiliations:** ^1^ Institute of Forest Sciences, The Islamia University, Bahawalpur, Pakistan; ^2^ Farmland Irrigation Research Institute, Chinese Academy of Agricultural Sciences, Xinxiang, Henan, China; ^3^ Institute of Environmental Studies, University of Karachi, Karachi, Pakistan; ^4^ Department of Forestry and Range Management, Bahauddin Zakariya University, Multan, Pakistan; ^5^ Department of Forestry, University of Sargodha, Sargodha, Pakistan; ^6^ Department of Botany, University of Karachi, Karachi, Pakistan; ^7^ Water Science and Environmental Engineering Research Center, College of Chemical and Environmental Engineering, Shenzhen University, Shenzhen, China

**Keywords:** phytoremediation, bioindicator, air pollution, soil pollution, antioxidant enzyme, reactive oxygen species

## Abstract

Soil and air pollution caused by heavy metals and limestone dust are prevalent in urban environments and they are an alarming threat to the environment and humans. This study was designed to investigate the changes in morphological and physiological traits of three urban tree species seedlings (*Bombax ceiba, Conocarpus lancifolius*, and *Eucalyptus camaldulensis*) under the individual as well as synergetic effects of heavy metal lead (Pb) and limestone dust toxicities. The tree species were grown under controlled environmental conditions with nine treatments consisting of three levels of dust (0, 10, and 20 g) and three levels of Pb contaminated water irrigation (0, 5, and 10 mg L^−1^). The results depicted that the growth was maximum in T_1_ and minimum in T_9_ for all selected tree species. *B. ceiba* performed better under the same levels of Pb and limestone dust pollution as compared with the other two tree species. The *B. ceiba* tree species proved to be the most tolerant to Pb and limestone pollution by efficiently demolishing oxidative bursts by triggering SOD, POD, CAT, and proline contents under different levels of lead and dust pollution. The photosynthetic rate, stomatal conductance, evapotranspiration rate, and transpiration rate were negatively influenced in all three tree species in response to different levels of lead and dust applications. The photosynthetic rate was 1.7%, 3.1%, 7.0%, 11.03%, 16.2%, 23.8%, 24.8%, and 30.7%, and the stomatal conductance was 5%, 10.5%, 23.5%, 40%, 50.01%, 61.5%, 75%, and 90.9%, greater in T_2_, T_3_, T_4_, T_5_, T_6_, T_7_, T_8_, and T_9_ plants of *B. ceiba*, respectively, as compared to T_1_. Based on the findings, among these three tree species, *B. ceiba* is strongly recommended for planting in heavy metal and limestone dust-polluted areas followed by *E. camaldulensis* and *C. lancifolius* due to their better performance and efficient dust and heavy metal-scavenging capability.

## Graphical Abstract

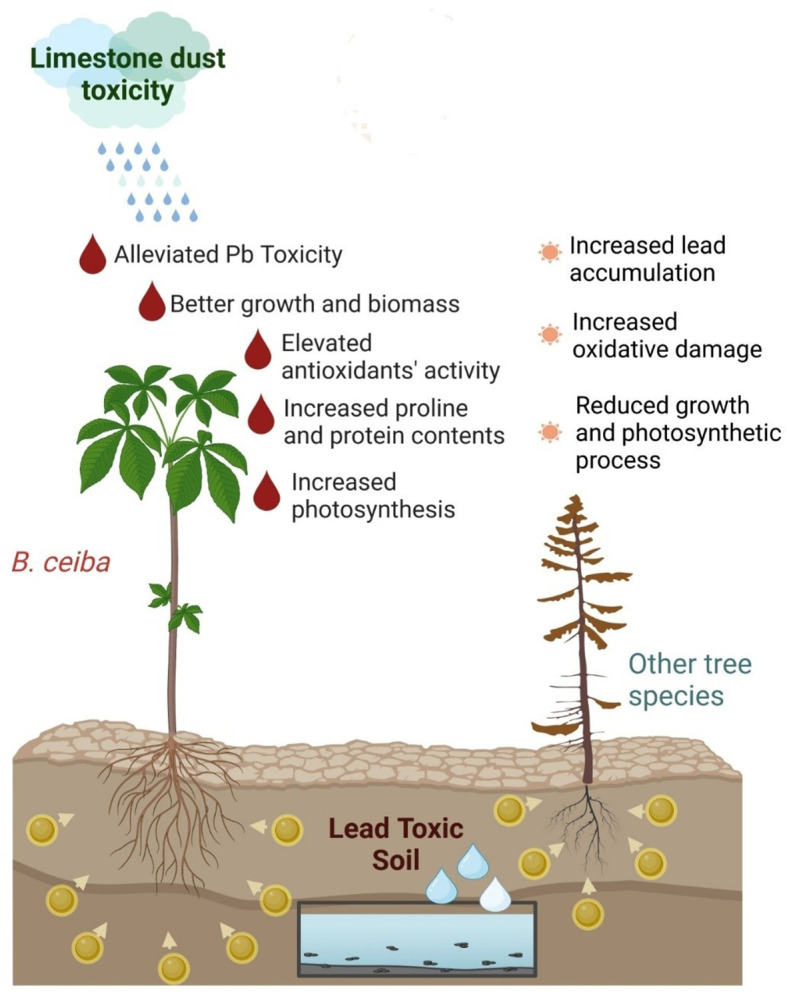

## 1 Introduction

The extensive alteration in the environmental conditions has caused environmental degradation (Patz et al., 2014; Xuebin et al., 2020). Previous studies have intensively discussed humans’ interaction with their surroundings and the influence of anthropogenic activities on the physical environment, either air, water, or soil (Ukaogo et al., 2020; Ur Rahman et al., 2021b). Anthropogenic activities including industrialization, burning fossil fuels, wastewater disposal, excessive chemical fertilizers, transportation, and urbanization negatively affect the environment by polluting the soil and the environment (Hussain et al., 2021; Kuppala, 2021; Yasir et al., 2021). Air pollution is one of the major public health threats worldwide, causing approximately 9 million casualties each year (Manisalidis et al., 2020). The current scenario of deforestation and the devastation of biota are recognized as the increasing cause of dust and soil pollution (Moore, 2009).

It is well known that air pollution causes adverse health effects (Tolinggi et al., 2014; Yasir et al., 2021). Limestone, calcareous sedimentary rock in nature, is considered as one of the most versatile minerals globally and used as raw material in various construction industries (Chaulya, 2003). It is generally believed that construction sites, including blasting, drilling, and transportation, are the primary factors that cause limestone dust production (Akande and Ifelola, 2011). Limestone dust adversely affects plant physiology and growth traits (Sett, 2017). Several studies reported the reduced production of chlorophyll pigments due to the shading effects of limestone dust deposition on leaves. Moreover, prevalent alkaline conditions are being reported due to the lime solubility in the cell sap, which causes chlorophyll damage and retardation of photosynthesis (Grantz et al., 2003). Along with restricted photosynthesis, the production of biomonitoring compounds such as starch and protein is reduced due to lime dust (Van Heerden et al., 2007).

Previous researchers have reported the potential tree species for the removal of limestone-oriented dust pollution (Panichev and McCrindle, 2004; Kończak et al., 2021). For instance, Szwed et al. (2021) reported that pine needles are decent bioindicators of limestone dust pollution due to the unique chemical composition. In another study, Łukowski et al. (2020) used *Betula pendula* Roth., *Quercus robur* L., and *Tilia cordata* Mill tree species to eliminate the particulate matter (PM) from the environment and reported that *B. pendula* was most effective in phytoextraction and improving the quality of PM-contaminated air. However, only a limited number of studies evaluated the phytoremediation potential of *Bombax ceiba*, *Eucalyptus camaldulensis*, and *Conocarpus lancifolius* against limestone dust pollution. Thus, considering this research gap, we conducted the present study to assess the phytoremedial potential of common urban tree species, i.e., *B. ceiba*, *E. camaldulensis*, and *C. lancifolius*, for limestone dust pollution.

Lead (Pb), a toxic trace metal, is a naturally occurring element in the soil (Nas and Ali, 2018). Anthropogenic activities including the intensive use of Pb-oriented products have led to the increase in Pb concentrations in urban soils (Reimann et al., 2012; Mitra et al., 2020). Owing to its durability and inertness, Pb does not decompose and remains in soils for a longer period of time (Fahr et al., 2013). Furthermore, Pb availability, solubility, and mobility are very low in plants because Pb precipitates with sulfates, phosphates, and other chemicals in the rhizosphere (Gratão et al., 2005; Fahr et al., 2013), although plant roots excrete different metabolites and exudates to adjust soil pH, restrict the dissolution procedure, and reduce the formation of Pb precipitation with organic complexes (Fahr et al., 2013). Under high Pb concentrations, limited cell division restricted root development and elongation (Mitra et al., 2020). Moreover, higher Pb concentrations resulted in protuberances, cell wall distention, and the development of root tissues (Alamri et al., 2018; Mitra et al., 2020). Pb causes oxidative stress, peroxidation, and membrane damage by producing intensive oxidants such as hydrogen peroxide (H_2_O_2_) and oxygen radicals (O_2_^−^) (Sharma and Dubey, 2005; Liu et al., 2010; Su et al., 2019). Plants elevate ROS toxicity by increasing the activities of antioxidant enzymes [i.e., superoxide dismutase (SOD), catalase (CAT), and peroxidase (POD)], non-enzymes/metabolites (i.e., ascorbate and glutathione), and osmolytes (including proline) (Gill and Tuteja, 2010).

In previous studies, tree plant species have been used to alleviate Pb toxicities in the soil and environment (Ali et al., 2013; Satpathy and Reddy, 2013; Xiao et al., 2018; Wu et al., 2021; Yasin et al., 2021); however, there are various research gaps in selecting the most suitable tree species based on their physiological responses against atmospheric and soil pollution. The hypothesis of this study is that exposure to limestone dust and lead pollution will negatively impact the ecophysiology of urban tree species. Because limestone dust and lead are the most common urban hazards in the country, the present study was therefore conducted to evaluate the phytoremediation potential of the most commonly existing urban tree species, i.e., *B. ceiba*, *E. camaldulensis*, and *C. lancifolius*, based on their physiological and biochemical responses under different levels of Pb and lime dust toxicities.

## 2 Materials and methods

### 2.1 Study area and experimental layout

**Figure 1:**
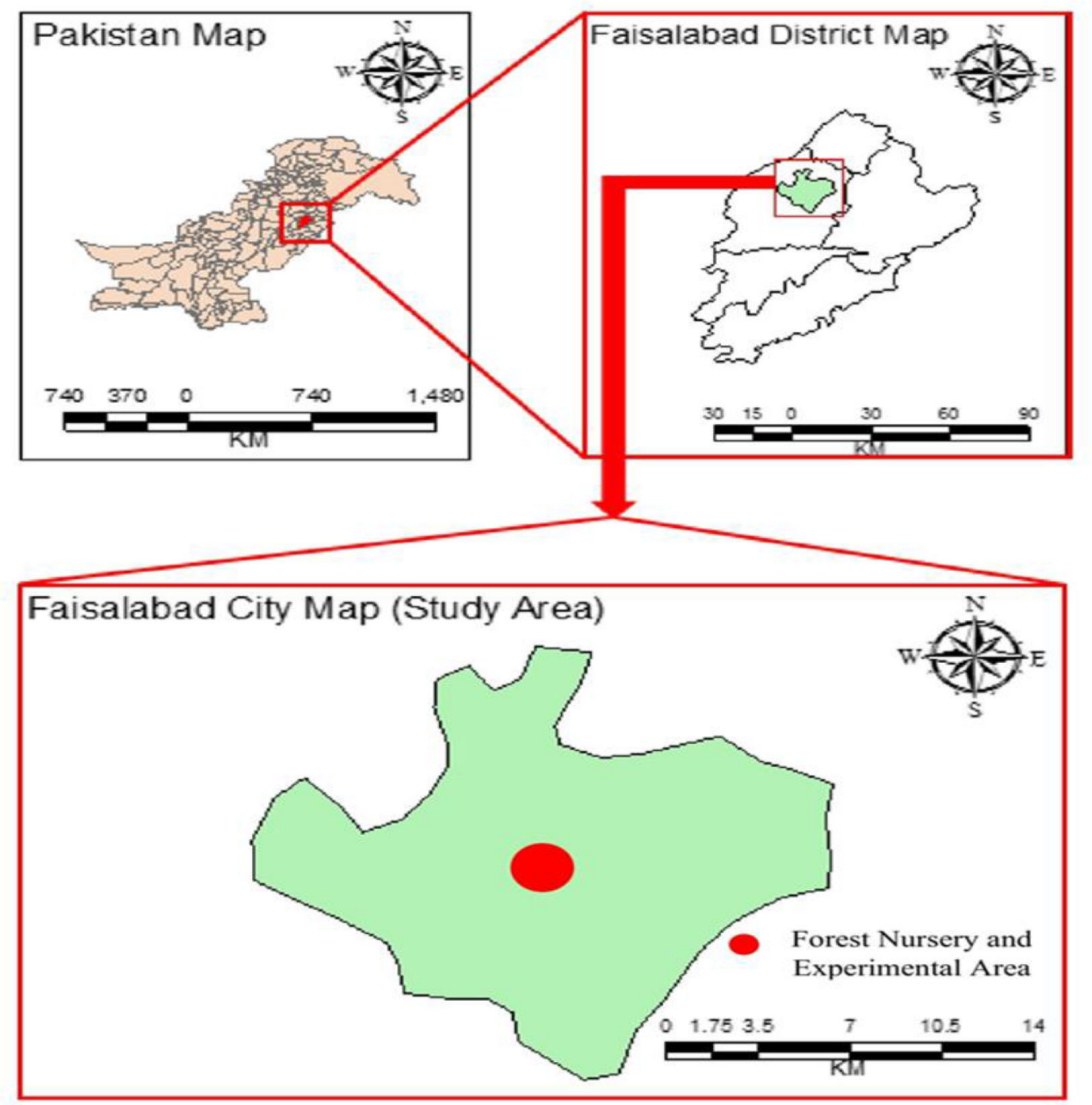
Graphical presentation of the study area.

The sandy loam soil used in this experiment was collected from the Forest Nursery and Experimental Area, Department of Forestry and Range Management, University of Agriculture, Faisalabad, Punjab province, Pakistan (see **Figure 1** for study area). It is located 31.4471° N, 73.0713° E, with an altitude of approximately 184 m (604 ft) above sea level. The well-sieved air-dried soil was used to fill the earthen pots and 8 kg soil was used in each pot. The 6-month-old healthy, disease-free (sterilized with 70% ethanol for 1 min and 5% sodium hypochlorite for 3 min), and same-sized seedlings of *E. camaldulensis* and *C. lancifolius* and stumps cutting of *B. ceiba*, taken from Punjab Forestry Research Institute (PFRI), Faisalabad, were transplanted into earthen pots where irrigation was supplied throughout the growing season. When the seedlings were established, three levels of limestone dust (0, 10, and 20 g L^−1^) and Pb (0, 5, and 10 mg L^−1^) were applied as treatments, which were arranged as follows: T_1_ (control), T_2_ (10 g dust), T_3_ (20 g dust), T_4_ (5 mg L^−1^ Pb), T_5_ (10 g dust + 5 mg L^−1^ Pb), T_6_ (20 g dust + 5 mg L^−1^ Pb), T_7_ (10 mg L^−1^ Pb), T_8_ (10 g dust + 10 mg L^−1^ Pb), and T_9_ (20 g dust + 10 mg L^−1^ Pb). A powder coating gun was used to apply limestone dust in the form of loosened powder on the leaf surface. Lead application was made through irrigation. We divided each lead and limestone dust concentration treatment into six sub-solutions to maintain the measured quantities according to the above-described treatments. The application of both limestone and Pb was into six splits with two applications per week. The experiment consisted of three replications and three plants per replication for one individual tree species. A total 243 plants were used for three tree species (3 species × 3 plants × 3 replicates × 9 treatments).

### 2.2 Data collection

#### 2.2.1 General characteristics of used species

The necessary details regarding the genus, family, and major characteristics of used tree species are mentioned in **Table 1**.

**Table 1:** The name of genus, family, and characteristics of tree species used in the present study.

Tree Species	Genus	Family	Characteristics
***Eucalyptus camaldulensis***	Eucalyptus	Myrtaceae	It is commonly known as the river red gum. It is well recognized as a highly adopting tree species with maximum seed germination, sustainable vegetative growth, and reproduction ability at early ages under diverse environmental conditions like heavy metal, saline, and drought stress. Generally, its height is up to 66 ft, but in rare cases, its height is noted up to 148 ft, without developing lignotuber.
***Bombax ceiba***	Bombax	Malvaceae	It is commonly known as a cotton tree. Its height is 20 m in general, but very old trees are noticed up to 60 m tall. The trunk and limb bear numerous conical spines, particularly when young, but get eroded when older. This tree species is also well-recognized for its phytoremediation ability of soil, water, and air pollutant due to its tall and straight trunk.
***Conocarpus lancifolius***	Conocarpus	Combretaceae	It is an evergreen tree, usually growing 10–20 m tall but exceptionally to 30 m. The bole can be 90 cm in diameter. This tree is used as a medicine and good quality wood and fuel source. Owing to its ability to withstand heavy metal, salt, and drought stresses, this tree is preferred to be planted in reafforestation missions.

#### 2.2.2 Stem height and stem diameter

The stem height was examined from the collar point to the apex of the entire tree by using a gauging tape. The stem diameter was measured using a digital caliper at the collar, which was marked beforehand. Two readings were taken at the collar point, diagonal to each other, to cover the circumferential variability, and the mean diameter was calculated for all individuals. These activities were performed two times, initially when seedling was established and finally before harvesting. The root and shoot sections of the harvested plants were weighed immediately by placing them into the paper bags by using a weighing balance (Electronic Scale JJ3000B). Finally, these samples were placed into an oven (DGH - 9202 series Thermal Electric Thermostat Drying Oven) at 74°C for 5 days until the constant weight and dry weight was recorded by using a weight balance.

#### 2.2.3 Tree biomass

The biomass partitioning was divided into the shoot biomass and root biomass, which were measured according to the given formulas.


Shoot biomass(%)=Shoot biomassTotal biomass×100

Root biomass(%)=Root biomassTotal biomass×100

Total biomass=Shoot biomass+Root biomass

#### 2.2.4 Gas exchange parameters

The physiological parameters were recorded by using the Infra-Red Gas Analyzer (IRGA). Leaf gas exchange measurements were taken from the second mature fully expanded leaf (during 9.00 a.m. to 11.00 a.m.) using a portable gas exchange system, CIRAS-3 (PP-Systems, Amesbury, MA 01913 USA). IRGA was used to measure stomatal conductance (gs, mmol m^2^ s^−1^), assimilation rate (A, μmol m^−2^ s^−1^), and transpiration rate (E, mmol m^−2^ s^−1^), and the following adjustments were made: carbon dioxide level of 380 ppm, leaf temperature of 30°C, and 52%–55% relative humidity inside the leaf chamber. Water use efficiency (WUE) was obtained by dividing the net CO_2_ assimilation and transpiration rates.

### 2.3 Photosynthetic parameter

Fully expanded leaves were considered to estimate the photosynthetic pigments such as chlorophyll and carotenoid contents. For that, 50 mg of fresh leaves was mashed in a mortar and pestle together with a modest number of sand particles and magnesium carbonate (MgCO_3_). Subsequently, the leaves were frozen in acetone (80%) to record the chlorophyll pigments and carotenoid contents. After cooling the suspension, the centrifuged solution (for 5 min at 4°C with a 5,000 *g* resolution) was used to analyze photosynthetic pigments through the spectrophotometry method (Ur Rahman et al., 2021a).

#### 2.3.1 Estimation of lead content

The dry ground material (5 g) was incubated in 5 ml of nitric acid for 12 h at room temperature (Wolf, 1982). The flasks were placed onto a hot electrical plate and roasted at 350°C until vapors began to form. This process was performed several times until the digesting material became clear and colorless in appearance. The translucent extract was filtered using a Whatman paper and increased the solution volume to 50 ml by adding distilled water.

An Atomic Absorption Spectrophotometer (Hitachi Polarized Zeeman AAS, Z-8200, Japan) at Hi-tech Laboratory, University of Agriculture, Faisalabad was used to calculate the Pb concentration. For that, the following settings were adjusted: 283.3 nm of wavelength, 7.5 mA of lamp current, 1.3 nm slit width, and 160 kPa oxidant gas pressure. A standard type of burner head with 7.5 mm height and 7 kPa fuel gas pressure was used in this process.

A commercially existing aqueous solution (1,000 ppm) was used to prepare the calibrated curve. Highly purified deionized water was used to reach working standards.

### 2.4 Estimation of dust load

Dust load was estimated using the pre-described method of Chaturvedi et al. (2013). Fully matured leaves were used for this purpose. Leaves were washed with distilled water and filtered through sieve paper. The wet sieve paper was first weighed, and then dried and weighed to measure the dust. Top pan electronic balance (Model - CAC-34, Contech Instruments Limited, Bengaluru, India) was used to weight the dust amount on leaves, by using the following equation:


$$W=W_{2}-W_{1}/A$$

Here, *W* indicates the dust particulates (g m^−2^), *W_1_* is the primary weight of sieve paper, *W*_2_ is the final weight of sieve paper along with dust particulates, and *A* is the total leaf area (m^2^), measured using the leaf area meter.

### 2.5 Extract preparation for biochemical analysis

To analyze the soluble protein content and antioxidant enzyme activities (i.e., SOD, CAT, and POD), extraction was made according to the method of Naqvi et al. (2011). For that, 0.5 g of leaves was ground in a mortar and pestle and extracted in 2 ml of 50 mM phosphate buffer. The extracts were filtered and centrifuged at 8,000 rpm for 10 min and later used for the measurement of antioxidant activities.

#### 2.5.1 Superoxide dismutase

Superoxide dismutase activity was measured by determining its capability to hinder the photo-reduction of Nitroblue tetrazolium (NBT), according to the described technique of Giannopolitis and Ries (1977) with some modifications (Ur Rahman et al., 2021c). The reaction solution containing 0.015 g of NBT in 17.5 ml of distilled water, 0.222 g of methionine in 15 ml of distilled water, 0.0132 g of riboflavin in 17.5 ml of distilled water, 0.0375 g of Triton-X in 17.5 ml of distilled water, and 0.2 molar buffer was used for estimating SOD activity. The tube containing the reaction solution was placed under a UV lamp before adding riboflavin for 15 min. The absorbance was recorded at 560 nm wavelength spectrophotometrically. The amount of SOD enzyme that inhibited 50% of NBT photo-reduction was considered one unit activity of SOD.

##### 2.5.1.1 Peroxidase

Peroxidase activity was calculated using the method of Ur Rahman et al. (2021a). The reaction solution contained 50 mM phosphate buffer (pH 5), 20 mM guaiacol, 40 mM H_2_O_2_, and 0.1 ml of enzyme extract. Changes in the absorbance of reaction solution were determined by using a spectrophotometer at a wavelength of 470 nm. An absorbance change of 0.01 units per minute was considered as one unit activity of POD.

##### 2.5.1.2 Catalase

Catalase activity was determined using the technique of Ur Rahman et al. (2021c). The reaction solution contained 1.9 ml of phosphate buffer (pH 7), 1 ml of 5.9 Mm, and 0.1 ml of enzyme extract. The absorbance by using the reaction mixture was taken at 240 nm at 20-s intervals. One unit of CAT activity was defined as an absorbance change of 0.01 units per minute. The activity of each enzyme was expressed on a protein basis, which was measured according to Bradford (1976).

##### 2.5.1.3 Determination of soluble protein

The soluble protein contents were determined according to the Bradford method (Bradford, 1976) with some modifications (Rahman et al., 2021). Fifty microliters of the sample was taken in a microcentrifuge tube in which 2 ml of Bradford reagent was added. The blank sample only contained Bradford reagent. Absorbance was taken at a wavelength of 595 nm. To quantify the protein contents, a standard curve containing different concentrations of bovine serum albumin (BSA) was used.

##### 2.5.1.4 Determination of proline

Proline contents were estimated by using the method of Ur Rahman et al. (2021a). Firstly, 3 g of sulfosalicylic acid was dissolved in 50 mM of PBS (pH.7.8) and made the final volume up to 100 ml. Approximately 40.93 ml of 85% H_3_PO_4_ was taken, and its volume was raised to 100 ml. After that, 60 ml of pure acetic acid was mixed in 40 ml of H_3_PO_4_ (6M) solution. After adding 2.5 g of Ninhydrin, the solution was heated at 700°C, and later stored at 40°C. The fresh leaf samples were cut into small pieces, and extract solution was made by using 5 ml of 3% sulfosalicylic acid. Later, solution was incubated in boiling water at 100°C for 10 min. The samples were cooled down using tap water, and then 1 ml supernatant was taken and supplemented with 1 ml of pure acetic acid and 1.5 ml of Ninhydrin. The solution was heated in a water bath (100°C) for 40 min. Later, 2.5 ml of toluene was added in the cooled sample. The samples were then allowed to settle down for several minutes. Finally, the supernatant was used for reading spectrophotometrically. Pure toluene was used as control.


Proline content (μg/gFW)=C×V/a×W

Here, *C* is the proline content calculated from the standard curve, *V* is the volume of solution used to extract proline (5 ml), *a* is the volume of supernatant used (1 ml), and *W* is the sample weight.

### 2.6 Statistical analysis

The data were subjected to variance analysis using complete randomized design (CRD) by using Statistix 8.1. Vertical bars on each column indicate ± SE (*n* = 3), each with three replicates. Means with different letters differ significantly at 5% probability.

## 3 Results

### 3.1 Growth traits

It was observed that tested tree species depicted a significant variation for growth traits: shoot fresh weight and dry weights, root fresh weight and dry weights, and stem height and stem diameter under different lead and limestone dust concentrations (**Table 2**). The interactive effect of these treatments showed a significant variation in growth traits of selected tree species. The observed results showed that the shoot fresh and dry weights, root fresh and dry weights, and stem height and stem diameter were maximum under control and they gradually decreased with the increase in Pb and limestone dust concentrations for all three tree species. For instance, the shoot fresh weight for *B. ceiba* was 2.4%, 5.8%, 10.4%, 13.6%, 15.2%, 17.6%, 23.3%, and 25.7% greater in control as compared to all corresponding treatments. Similarly, shoot dry weight for *B. ceiba* was 3.7%, 6.9%, 8.5%, 12.5%, 15.0%, 20.4%, 29.6%, and 34.3% greater in control as compared to all corresponding treatments (**Table 2**).

**Table 2:** Effect of different levels of limestone dust and lead toxicities on root fresh weight (RFW), root dry weight (RDW), shoot fresh weight (SFW), shoot dry weight (SDW), stem diameter (SD), and stem height (SH) of *B. ceiba*, *E. camaldulensis*, and *C. lancifolius*, respectively.

Treatments	*Bombax ceiba*					
	S.H. (cm)	S.D. (mm)	SFW (g plant^−1^)	SDW (g plant^−1^)	RFW (g plant^−1^)	RDW (g plant^−1^)
**T_1_**	73.7 ± 3.01^a^	25.1 ± 0.94^a^	144.4 ± 3.71^a^	85.8 ± 2.33^a^	95.8 ± 2.56^a^	63.8 ± 2.45^a^
**T_2_**	72.1 ± 2.76^ab^	23.8 ± 0.50^b^	141.0 ± 2.98^b^	82.7 ± 1.93^b^	93.3 ± 2.44ab	61.6 ± 3.44^b^
**T_3_**	69.1 ± 3.22^cd^	22.7 ± 0.37b^c^	136.4 ± 4.01^c^	80.2 ± 3.11^c^	90.1 ± 1.76^bc^	58.2 ± 2.88^c^
**T_4_**	67.4 ± 2.00^de^	21.6 ± 0.99^ce^	130.8 ± 4.22^d^	79.1 ± 2.00^cd^	88.2 ± 3.65^cd^	55.7 ± 3.00^d^
**T_5_**	66.2 ± 2.98^ef^	20.6 ± 1.09^ef^	127.1 ± 2.88^e^	76.3 ± 1.49^ef^	83.9 ± 2.89e^f^	53.1 ± 2.87^e^
**T_6_**	63.3 ± 1.19^gh^	18.9 ± 0.22^g^	125.3 ± 3.78^ef^	74.6 ± 2.31f^g^	80.6 ± 2.01^fg^	51.7 ± 1.89^e^
**T_7_**	61.9 ± 3.01^hi^	17.3 ± 0.83^hi^	122.8 ± 3.81^fg^	71.2 ± 1.65^hi^	77.4 ± 3.02^g-i^	47.0 ± 0.86^g^
**T_8_**	60.1 ± 1.39^ij^	16.7 ± 0.61^hj^	117.1 ± 1.66^h-j^	66.2 ± 1.72^kl^	74.0 ± 2.77^ij^	45.1 ± 2.91^gh^
**T_9_**	58.7 ± 2.99^j^	15.6 ± 0.51^j-l^	114.9 ± 2.77^i^	63.9 ± 1.56l^-n^	71.1 ± 2.53^j-l^	41.8 ± 2.34^i^
*Eucalyptus camaldulensis*						
**T_1_**	71.0 ± 2.09^bc^	22.2 ± 0.55^cd^	120.3 ± 3.01^gh^	77.6 ± 3.01d^e^	87.2 ± 3.00^ce^	56.2 ± 3.01^cd^
**T_2_**	68.4 ± 1.76^d^	21.4 ± 0.61^de^	108.8 ± 2.91^j^	73.3 ± 1.45^gh^	85.2 ± 2.34^de^	52.4 ± 2.45^e^
**T_3_**	64.3 ± 2.62^fg^	20.6 ± 0.71^ef^	103.2 ± 2.43^k^	69.1 ± 2.78^ij^	79.6 ± 2.09^gh^	49.4 ± 1.87^f^
**T_4_**	62.2 ± 1.84^h^	19.1 ± 0.42^g^	97.2 ± 3.22^lm^	64.1 ± 3.00l^m^	77.1 ± 1.83^jk^	43.2 ± 1.67^hi^
**T_5_**	58.7± 2.76^i^	17.6 ± 0.71^h^	93.4 ± 2.11^no^	61.7 ± 2.14^n^	70.3 ± 2.76^kl^	39.3 ± 2.9^8i^
**T_6_**	54.3 ± 2.35^k^	16.2 ± 0.50i^-k^	89.2 ± 3.61^p^	58.0 ± 2.99^op^	68.3 ± 1.93^l-n^	36.2 ± 3.22^kl^
**T_7_**	53.7 ± 2.33^kl^	15.3 ± 0.33^k-m^	82.2 ± 1.55^qr^	56.3 ± 1.99^pq^	66.0 ± 3.21^mo^	35.5 ± 1.66^kl^
**T_8_**	51.8 ± 3.44l^m^	14.2 ± 0.49^mn^	79.1 ± 2.45^rs^	53.9 ± 2.09^rs^	63.8 ± 1.92^op^	31.9 ± 2.11^mn^
**T_9_**	51.4 ± 2.43^m^	13.5 ± 0.82^no^	76.1 ± 3.44^st^	50.1 ± 2.00^tu^	59.9 ± 3.00^q^	29.8 ± 3.10^no^
*Conocarpus lancifolius*						
**T_1_**	61.9 ± 3.44^hi^	19.8 ± 0.73^fg^	105.2 ± 2.45^k^	67.9 ± 2.19^jk^	76.2 ± 2.54^hi^	42.7 ± 2.99^i^
**T_2_**	53.4 ± 2.99^kl^	17.1 ± 0.39^hi^	99.3 ± 3.22l	62.3 ± 2.01^mn^	69.4 ± 2.91^k-m^	37.2 ± 1.76^k^
**T_3_**	52.6 ± 2.99^lm^	15.8 ± 0.78j^-l^	95.1 ± 3.24^mn^	59.1 ± 2.91°	65.3 ± 3.11^no^	34.6 ± 2.55^l^
**T_4_**	49.1 ± 2.20^n^	14.7 ± 0.67l^m^	90.4 ± 2.99^op^	55.6 ± 2.67^qr^	60.3 ± 1.45^pq^	32.2 ± 3.01^m^
**T_5_**	48.0 ± 2.87^n^	13.4 ± 0.56^no^	84.6 ± 3.09^q^	51.7 ± 2.22^st^	57.7 ± 1.87^qr^	28.6 ± 2.25^op^
**T_6_**	45.9 ± 2.11°	12.5 ± 0.98^op^	80.1 ± 3.45^r^	48.4 ± 3.01^u^	55.9 ± 1.76^r^	27.5 ± 1.87^pq^
**T_7_**	44.0 ± 3.00^op^	11.9 ± 0.67^pq^	73.1 ± 2.87^t^	45.1 ± 1.70^v^	51.4 ± 2.44^s^	25.8 ± 2.43^q^
**T_8_**	42.3 ± 2.88^pq^	10.9 ± 0.33^qr^	69.4 ± 2.01^u^	43.2 ± 1.67^vw^	47.7 ± 2.99^t^	23.5 ± 2.17^r^
**T_9_**	41.7 ± 1.67^q^	10.6 ± 0.69^r^	63.6 ± 2.85^v^	41.0 ± 2.76^w^	44.7 ± 1.26^t^	22.0 ± 2.09^r^

The values are expressed as the mean ± SD (n = 3). The different letters within a column indicate significant differences within treatments at p< 0.05.

The root fresh weight was maximum in T_1_ for all tree species—*B. ceiba* (95.8* g*), *E. camaldulensis* (87.2 g), and *C. lancifolius* (76.2 g)—and minimum in T_9_ (71.1 g, 59.9 g, and 44.7 g, respectively). Similarly, root dry weight for *B. ceiba* was 3.5%, 9.6%, 14.5%, 19.9%, 23.4%, 35.5%, 41.4%, and 52.6% lower in all corresponding treatments as compared to control (T_1_). The stem height and diameter were maximum in T_1_ for all tree species—*B. ceiba* (73.7* cm* and 25.1 mm), *E. camaldulensis* (71 cm and 22.2 mm), and *C. lancifolius* (61.9 cm and 19.8 mm)—and minimum in T_9_. The general trend of species response under different Pb and limestone dust stress for the shoot fresh and dry weight was *B. ceiba* > *E. camaldulensis* > *C. lancifolius* (**Table 2**).

### 3.2 Physiological traits

#### 3.2.1 Photosynthetic pigments

**Figure 2:**
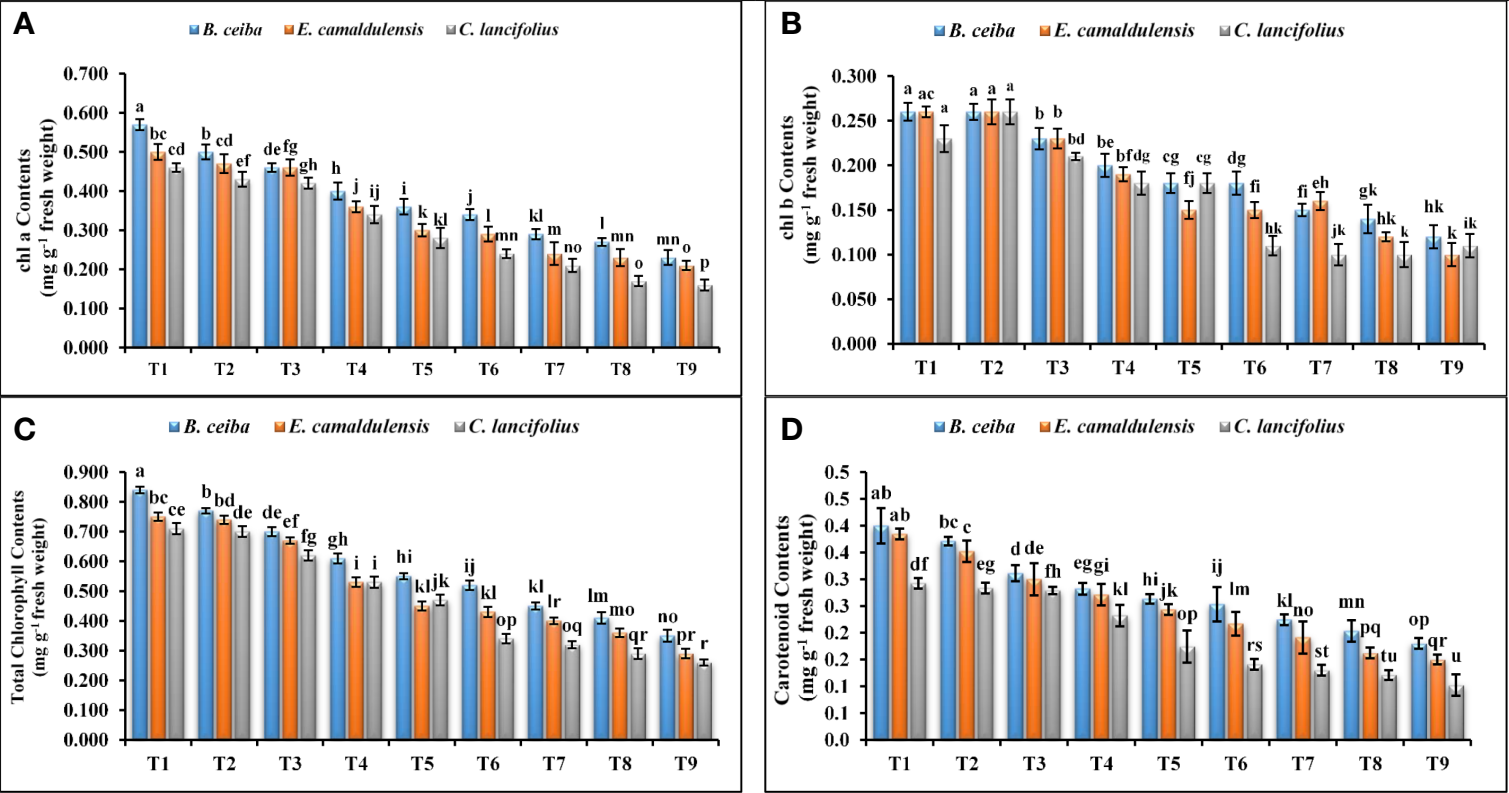
Effect of different levels of limestone dust and lead toxicities on **(A)** photosynthetic a, **(B)** photosynthetic b, **(C)** carotenoids, and **(D)** total chlorophyll in the fresh leaves of *B. ceiba*, *E. camaldulensis*, and *C. lancifolius*. FW represents the fresh weight of leaf samples and the different letters within a column indicate significant differences within treatments at *p*< 0.05.

The findings of the current study revealed that tested tree species depicted a significant variation for chlorophyll a, b, total chlorophyll, and carotenoid contents under different Pb and limestone dust concentrations (**Figures 2A–D**). The interactive effect of tree species and Pb and limestone dust concentrations also showed a highly significant variation for these traits. Significantly maximum values of these traits were recorded for control treatment (**Figure 2**). For instance, the chlorophyll a concentration for *B. ceiba* was 14%, 24.0%, 42.5%, 58.3%, 67.6%, 96.6%, 111.1%, and 147.83%; the chlorophyll b concentration was 0.4%, 13.04%, 30%, 44.4%, 44.4%, 73.3%, 85.7%, and 116.6%; the total chlorophyll concentration was 7.1%, 16.8%, 25%, 34.3%, 40.6%, 47.5%, 57.9%, and 63.6%; and the carotenoid contents were 8.1%, 29.03%, 42.8%, 53.8%, 60%, 81.8%, 100%, and 122.2% greater in the control treatment (T_1_) as compared to the corresponding treatments T_2_, T_3_, T_4_, T_5_, T_6_, T_7_, T_8_, and T_9_, respectively. A similar trend was recorded in the remaining tree species. The species response under different Pb and limestone dust stress for chlorophyll a, chlorophyll b, total chlorophyll, and carotenoids contents was *B. ceiba* > *E. camaldulensis* > *C. lancifolius*.

#### 3.2.2 Photosynthetic rate

**Figure 3:**
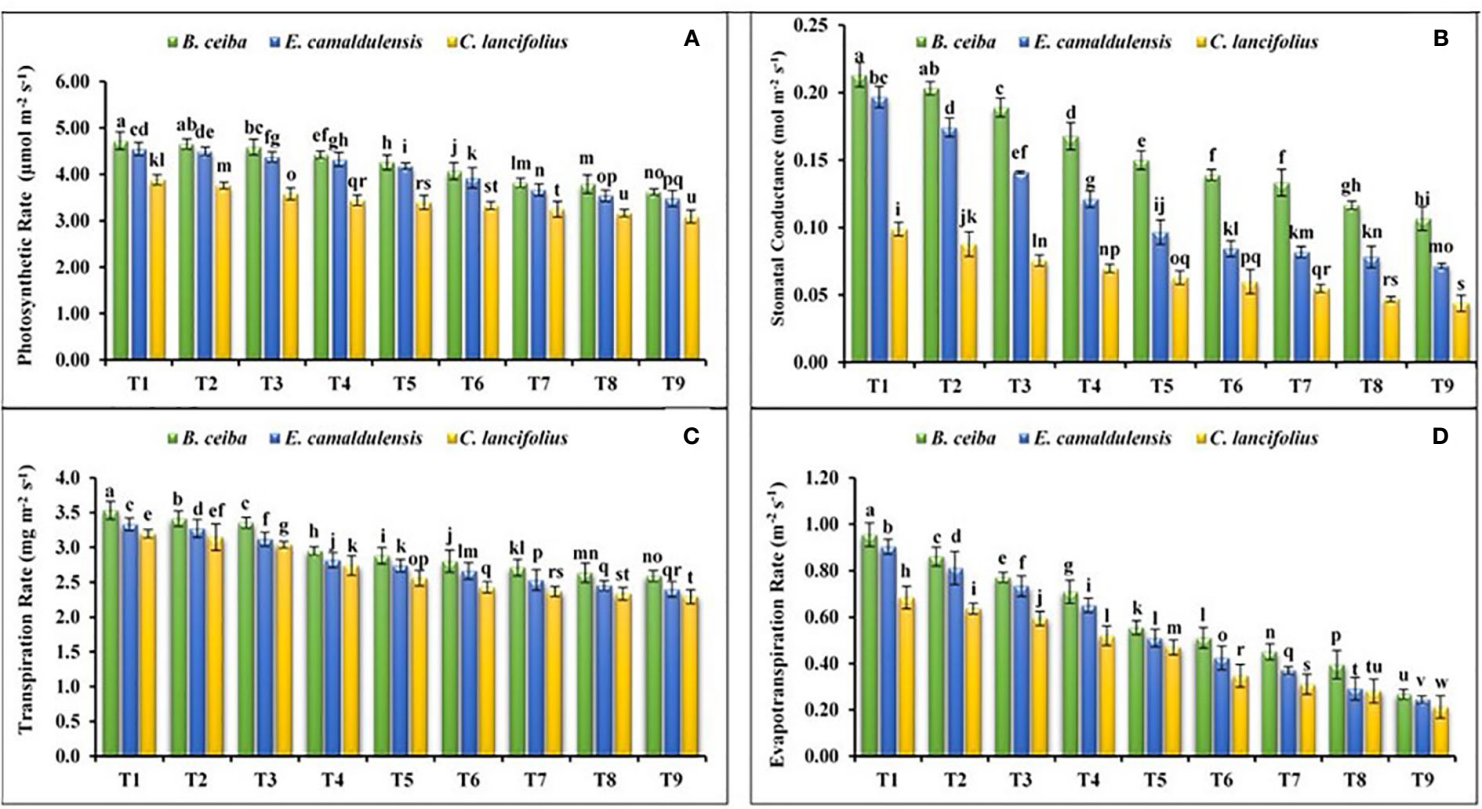
Effect of different levels of limestone dust and lead toxicities on **(A)** photosynthetic rate, **(B)** stomatal conductance, **(C)** transpiration rate, and **(D)** evapotranspiration rate of *B ceiba*, *E camaldulensis*, and *C lancifolius*. The different letters within a column indicate significant differences within treatments at *p*< 0.05.

The findings of the current study revealed that tested tree species depicted a significant variation for the photosynthetic rate under different Pb and limestone dust concentrations (**Figure 3A**). The interactive effect of various tree species and different levels of Pb and limestone dust concentrations also showed a significant variation in the photosynthetic rate. Among tree species, the photosynthetic rate for *B. ceiba* was greater under all treatments as compared to *E. camaldulensis* and *C. lancifolius*. The maximum concentration of photosynthetic rate was recorded in control (T_1_) treatment compared to the other treatments for all tested tree species. For instance, the photosynthetic rate for *B. ceiba* was 1.7%, 3.1%, 7.0%, 11.03%, 16.2%, 23.8%, 24.8%, and 30.7% greater in the control (T_1_) as compared to T_2_, T_3_, T_4_, T_5_, T_6_, T_7_, T_8_, and T_9_, respectively. A similar trend was recorded for the remaining two tree species. Moreover, species response under different Pb and limestone dust stress for the photosynthetic rate was *B. ceiba* > *E. camaldulensis* > *C. lancifolius*.

#### 3.2.3 Stomatal conductance

Data revealed that all tested tree species depicted a significant variation for the stomatal conductance under different Pb and limestone dust concentrations (**Figure 3B**). The interactive effect of various tree species and different Pb and limestone dust concentrations was also significant for the stomatal conductance. The maximum stomatal conductance was recorded in control (T_1_) treatment compared to the remaining treatments for all tested tree species. For instance, the stomatal conductance for *B. ceiba* was 5%, 10.5%, 23.5%, 40%, 50.01%, 61.5%, 75%, and 90.9% greater in control (T_1_) as compared to T_2_, T_3_, T_4_, T_5_, T_6_, T_7_, T_8_, and T_9_, respectively. The same trend was recorded for the remaining two tree species. Moreover, the species response under different Pb and limestone dust stress for the stomatal conductance was *B. ceiba* > *E. camaldulensis* > *C. lancifolius*.

#### 3.2.4 Transpiration rate

Recoded data revealed that all tree species depicted a significant variation for the transpiration rate under different Pb and limestone dust concentrations (**Figure 3C**). The interactive effect of various tree species and different Pb and limestone dust concentrations was also significant for transpiration rate. The maximum transpiration rate in all tested species was recorded in control (T_1_) treatment. For instance, the transpiration rate for *B. ceiba* was 2.9%, 2.9%, 16.7%, 20.7%, 25.01%, 29.6%, 34.6%, and 34.9% greater in control (T_1_) as compared to T_2_, T_3_, T_4_, T_5_, T_6_, T_7_, T_8_, and T_9_, respectively. A similar trend was recorded for the remaining tree species. Moreover, the species response under different Pb and limestone dust stress for the photosynthetic rate was *B. ceiba* > *E. camaldulensis* > *C. lancifolius*.

#### 3.2.5 Evapotranspiration rate

Data revealed that the selected tree species depicted a significant variation in the evapotranspiration rate under different Pb and limestone dust concentrations (**Figure 3D**). The interactive effect of various tree species and different Pb and limestone dust concentrations was also significant for the evapotranspiration rate. The maximum evapotranspiration rate was recorded in control (T_1_) treatment compared to the remaining corresponding treatments for all tested tree species. For instance, the stomatal conductance for *B. ceiba* was 10.5%, 23.4%, 33.8%, 72.7%, 86.3%, 111.1%, 143.6%, and 251.8.9% greater in control (T_1_) as compared to T_2_, T_3_, T_4_, T_5_, T_6_, T_7_, T_8_, and T_9_, respectively. A similar trend was recorded for the remaining two tree species. Moreover, the trend of species response under different Pb and limestone dust stress for the photosynthetic rate was *B. ceiba* > *E. camaldulensis* > *C. lancifolius*.

#### 3.2.6 Water potential (ψw), osmotic potential (ψs), and pressure potential (ψp)

**Figure 4:**
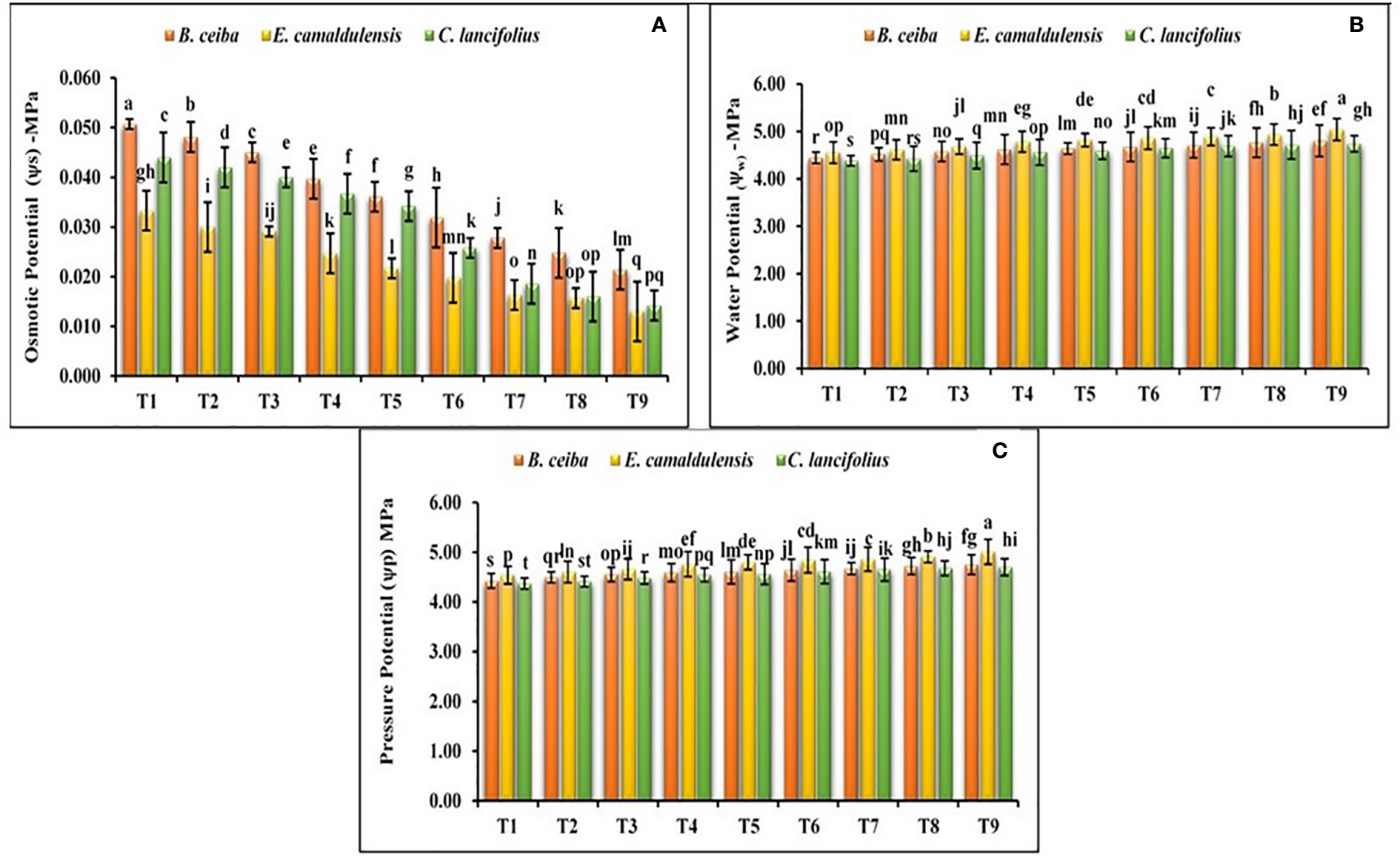
Effect of different levels of limestone dust and lead toxicities on **(A)** osmotic potential, **(B)** water potential, and **(C)** pressure potential of *B ceiba, E camaldulensis*, and *C lancifolius*. The different letters within a column indicate significant differences within treatments at *p*< 0.05.

The findings of the current study revealed that all selected tree species depicted a significant variation for the water potential, osmotic potential, and pressure potential under different Pb limestone dust concentrations (**Figures 4A–C**). The interactive effect of various tree species and different Pb and limestone dust concentrations was also significant for the water potential. Water potential in all tree species under all treatments was in the range of −4.39/−5.04 MPa. Among the tested tree species, water potential for *C. lancifolius* was greater under all treatments followed by *B. ceiba* and *E. camaldulensis*. The species response under different Pb and limestone dust stress for the water potential was *C. lancifolius* > *B. ceiba* > *E. camaldulensis*; for osmotic potential, it was *E. camaldulensis* > *C. lancifolius* > *B. ceiba*; and for pressure potential, it was *E. camaldulensis* > *B. ceiba* > *C. lancifolius*. Under different Pb and limestone dust concentrations, for water, osmotic, and pressure potential, treatments were ordered as T_9_ > T_8_ > T_7_ > T_6_ > T_5_ > T_4_ > T_3_ > T_2_ > T_1_ (**Figures 4A–C**).

### 3.3 Enzymatic antioxidants

Recorded data revealed that all tree species depicted a significant variation for enzymatic antioxidants under different Pb and limestone dust concentrations (**Table 3**). The interactive effect of various tree species and different Pb and limestone dust concentrations was also significant for CAT, POD, and SOD activities. The observed results showed that CAT, SOD, and POD activities in all tested species were minimum under control treatment. The activities of these antioxidants were gradually increased with the increase in Pb and limestone dust concentrations. The maximum concentration of stomatal conductance was recorded in the T_9_ treatment compared to the remaining corresponding treatments for all observed tree species. For instance, CAT activity for *B. ceiba* was 3.3%, 9.4%, 12.1%, 14.7%, 19.4%, 19.4%, 21.6%, and 23.6%; SOD activity was 4.1%, 7.1%, 9.5%, 11.1%, 12.2%, 14.8%, 16.8%, and 19.5%; and POD activity was 4.3%, 6.4%, 8.3%, 10.2%, 12%, 15.4%, 18.5%, and 18.7% greater in T_2_, T_3_, T_4_, T_5_, T_6_, T_7_, T_8_, and T_9_ treatment, respectively as compared to control (T_1_). A similar trend was recorded for other species. The species response under different Pb and limestone dust concentrations for the photosynthetic rate was *B. ceiba* > *E. camaldulensis* > *C. lancifolius*.

**Table 3:** Effect of different levels of limestone dust and lead toxicities on protein, CAT, SOD, POD, and proline contents of *B. ceiba*, *E. camaldulensis*, and *C. lancifolius*, respectively.

Treatments	*Bombax ceiba*				
	Protein (mg g^−1^ FW)	CAT (U mg^−1^ protein)	SOD (U mg^−1^ protein)	POD (U mg^−1^ protein)	Proline (µmol g^−1^ FW)
**T_1_**	1.31 ± 0.08^a^	2.85 ± 0.11°	292.3 ± 6.77^k^	4.42 ± 0.43^i^	0.58 ± 0.022^j^
**T_2_**	1.29 ± 0.04^a^	2.97 ± 0.21^n^	305.1 ± 5.98^i^	4.59 ± 0.23^h^	0.63 ± 0.034^i^
**T_3_**	1.26 ± 0.07^b^	3.23 ± 0.09^ij^	314.8 ± 9.77^g^	4.68 ± 0.11^g^	0.65 ± 0.039^i^
**T_4_**	1.22 ± 0.05^c^	3.31 ± 0.15^fg^	322.9 ± 8.67^f^	4.79 ± 0.17^f^	0.69 ± 0.029^f^
**T_5_**	1.18 ± 0.06^d^	3.45 ± 0.16^e^	328.9 ± 6.88^e^	4.92 ± 0.22^e^	0.77 ± 0.056^cd^
**T_6_**	1.15 ± 0.02^de^	3.56 ± 0.18^d^	333.1 ± 12^d^	5.01 ± 0.19^d^	0.79 ± 0.041^bc^
**T_7_**	1.14 ± 0.03^e^	3.61 ± 0.22^c^	343.2 ± 9.67^c^	5.18 ± 0.33^c^	0.80 ± 0.019^b^
**T_8_**	1.10 ± 0.04^f^	3.67 ± 0.31^b^	351.2 ± 11.34^b^	5.35 ± 0.13^b^	0.83 ± 0.044^b^
**T_9_**	1.02 ± 0.09^h^	3.76 ± 0.27^a^	363.3 ± 10.78^a^	5.41 ± 0.32^a^	0.87 ± 0.052^a^
*Eucalyptus camaldulensis*					
**T_1_**	0.87 ± 0.06^jk^	2.49 ± 0.17^st^	225.1 ± 8.99^u^	3.03 ± 0.21^w^	0.44 ± 0.047^n^
**T_2_**	0.79 ± 0.10^m^	2.60 ± 0.11^r^	239.8 ± 11.11^r^	3.19 ± 0.34^u^	0.47 ± 0.032^m^
**T_3_**	0.68 ± 0.09°	2.79 ± 0.17^p^	248.1 ± 10.76^pq^	3.36 ± 0.28^s^	0.51 ± 0.026^m^
**T_4_**	0.63 ± 0.04^p^	2.96 ± 0.14^n^	259.6 ± 7.44°	3.56 ± 0.31^p^	0.56 ± 0.045^j^
**T_5_**	0.58 ± 0.03^q^	3.12 ± 0.11^l^	266.1 ± 9.65^n^	3.69 ± 0.33^n^	0.66 ± 0.021f^-h^
**T_6_**	0.55 ± 0.08^r^	3.19 ± 0.13^jk^	277.2 ± 11.23^m^	3.83 ± 0.25^m^	0.67 ± 0.08^fg^
**T_7_**	0.53 ± 0.05^r^	3.25 ± 0.20^hi^	292.9 ± 13.87^k^	3.94 ± 0.11^l^	0.72 ± 0.034^e^
**T_8_**	0.48 ± 0.09^s^	3.31 ± 0.26^gh^	297.2 ± 7.78^i^	4.02 ± 0.39^k^	0.75 ± 0.028^d^
**T_9_**	0.45 ± 0.05^t^	3.37 ± 0.15^f^	310.3 ± 9.65^h^	4.17 ± 0.19^j^	0.77 ± 0.031^cd^
*Conocarpus lancifolius*					
**T_1_**	1.17 ± 0.09^d^	2.36 ± 0.19^u^	211.8 ± 10.76^w^	2.88 ± 0.19^y^	0.31 ± 0.071^p^
**T_2_**	1.07 ± 0.06^g^	2.47 ± 0.14^t^	218.7 ± 11.6^v^	2.98 ± 0.17^x^	0.34 ± 0.033°
**T_3_**	0.93 ± 0.09^i^	2.53 ± 0.10^s^	229.2 ± 9.01^t^	3.10 ± 0.33^v^	0.38 ± 0.059°
**T_4_**	0.88 ± 0.08^i^	2.62 ± 0.24^r^	234.7 ± 14.67^s^	3.16 ± 0.28^u^	0.42 ± 0.026^n^
**T_5_**	0.84 ± 0.03^kl^	2.73 ± 0.30^q^	246.9 ± 12.88^q^	3.27 ± 0.41^t^	0.47 ± 0.031^m^
**T_6_**	0.82 ± 0.04^l^	2.86 ± 0.35°	251.6 ± 11.08^p^	3.41 ± 0.13^r^	0.49 ± 0.045^m^
**T_7_**	0.79 ± 0.07^m^	2.94 ± 0.11^n^	258.3 ± 13.12°	3.50 ± 0.18^q^	0.53 ± 0.031^k^
**T_8_**	0.77 ± 0.03^m^	3.08 ± 0.19^m^	273.6 ± 7.18^m^	3.59 ± 0.25^p^	0.59 ± 0.043^j^
**T_9_**	0.73 ± 0.06^n^	3.17 ± 0.23^k^	284.1 ± 10.11^l^	3.65 ± 0.30°	0.64 ± 0.031^hi^

CAT, SOD, POD, and FW represent catalase, superoxide dismutase, peroxidase, and fresh weight, respectively. The values are expressed as the mean ± SD (n = 3). The different letters within a column indicate significant differences between the treatments at p< 0.05.

### 3.4 Non-enzymatic antioxidants

Recorded data revealed that all tested tree species depicted a significant variation for non-enzymatic antioxidant under different Pb and limestone dust concentrations (**Table 3**). The interactive effect of these treatments was also significant for protein and proline contents. Protein contents of three species were maximum under control, and gradually decreased with the increase in concentrations of Pb and limestone dust. For instance, proline concentration for *B. ceiba* was 7.9%, 10.8%, 15.9%, 24.7%, 27.5%, 27.5%, 29.3%, and 32.5% greater, and protein concentration for *B. ceiba* was 1.6%, 3.9%, 7.4%, 11.0%, 13.9%, 14.9%, 19.1%, and 28.4% smaller in T_2_, T_3_, T_4_, T_5_, T_6_, T_7_, T_8_, and T_9_, respectively, as compared to control (T_1_). Among tree species, protein and proline contents for *B. ceiba* were greater under all treatments followed by *E. camaldulensis* and *C. lancifolius*. The species response under different Pb and limestone dust stress for the protein contents was *B. ceiba* > *C. lancifolius* > *E. camaldulensis.*

### 3.5 Pb and dust load in subjected tree species

Data revealed that all selected tree species depicted a significant variation for dust load in leaves and Pb concentrations in different organs under different levels of Pb and dust toxicities (**Table 4**). Pb contents in the three selected tree species were maximum in *B. ceiba*, followed by *E. camaldulensis* and *C. lancifolius*. A similar trend was recorded for limestone dust treatments. For instance, Pb concentration in the root samples of *B. ceiba* was 15.3%, 4.4%, 16.8%, 13.8%, 21.9%,5.9%, 8.6%, 7.3%, and 8.4% higher than that of *E. camaldulensis*, and was 27.3%, 18.5%, 30.4%, 19.4%, 38.9%, 32.6%, 27.3%, 24.4%, and 25.6% higher than that of *C. lancifolius* in all corresponding treatments: T_1_, T_2_, T_3_, T_4_, T_5_, T_6_, T_7_, T_8_, and T_9_, respectively. A similar trend was recorded in both shoot and leaves of all studied tree species. For limestone dust load in leaves, the maximum concentration of dust was recorded in *B. ceiba*, followed by *E. camaldulensis* and *C. lancifolius*. For instance, the limestone dust concentration in *B. ceiba* was 18.1%, 20.7%, 59.3%, 21.2%, 16.5%, 48.9%, 5.4%, 19.3%, and 39.8% higher than that of *E. camaldulensis* and was 45.1%, 52.9%, 67.0%, 36.8%, 37.6%, 51.7%, 24.8%, 38.4%, and 60.3% higher than that of *C. lancifolius* in the corresponding treatments: T_1_, T_2_, T_3_, T_4_, T_5_, T_6_, T_7_, T_8_, and T_9_, respectively (**Table 4**).

**Table 4:** The concentration of Pb in the roots, shoots, and leaves, and the dust load in the leaves of *Bombax ceiba*, *Eucalyptus camaldulensis*, and *Conocarpus lancifolius*.

Treatments	*Bombax ceiba*			
	Root Pb Conc. mg kg^−1^ DW	Shoot Pb Conc. mg kg^−1^ DW	Leaves Pb Conc. mg kg^−1^ DW	Dust Load on Leaves mg cm^−2^
**T_1_**	0.91 ± 0.15^k^	0.84 ± 0.17^l^	0.59 ± 0.13^k^	8.66 ± 2.24^m^
**T_2_**	0.96 ± 0.18^k^	0.89 ± 0.15^l^	0.64 ± 0.12^k^	51.50 ± 4.82^de^
**T_3_**	1.14 ± 0.10^kl^	1.06 ± 0.29^lm^	0.76 ± 0.09^kl^	76.82 ± 5.74^b^
**T_4_**	6.15 ± 0.55^h^	5.25 ± 0.41^i^-^k^	4.18 ± 0.26^gh^	8.81 ± 2.48^kl^
**T_5_**	7.36 ± 0.56^g^	5.80 ± 0.43^hi^	4.69 ± 0.55^fg^	52.30 ± 5.66^de^
**T_6_**	8.09 ± 0.89^g^	6.14 ± 0.58^h^	5.05 ± 0.43^f^	80.78 ± 5.89^ab^
**T_7_**	11.67 ± 1.57^c^	10.72 ± 1.06^a-c^	8.17 ± 0.89^bc^	9.01 ± 2.26^k^
**T_8_**	12.44 ± 1.57^ab^	10.92 ± 0.84^ab^	8.48 ± 0.87^b^	55.07 ± 3.45^d^
**T_9_**	12.83 ± 1.53^a^	11.46 ± 1.05^a^	9.10 ± 1.34^a^	83.17 ± 4.82^a^
*Eucalyptus camaldulensis*				
**T_1_**	0.79 ± 0.10^k^	0.77 ± 0.18l	0.46 ± 0.10^k^	7.33 ± 1.83^mn^
**T_2_**	0.92 ± 0.12^k^	0.88 ± 0.11l	0.57 ± 0.11^k^	42.67 ± 4.83^gh^
**T_3_**	0.98 ± 0.11^k^	0.95 ± 0.13l^m^	0.62 ± 0.21^k^	48.22 ± 5.47^ef^
**T_4_**	5.40 ± 0.25^ij^	4.74 ± 0.89^k^	3.49 ± 0.51^ij^	7.27 ± 1.96^mn^
**T_5_**	6.03 ± 1.22^g^	4.84 ± 0.76^jk^	3.89 ± 0.70^hi^	44.91 ± 4.12^fg^
**T_6_**	7.64 ± 1.09^d^	5.56 ± 0.44^h-j^	3.99 ± 0.59^hi^	54.23 ± 4.58^d^
**T_7_**	10.74 ± 1.42^c^	9.45 ± 1.08^de^	7.12 ± 0.70^d^	8.55 ± 1.50^m^
**T_8_**	11.59 ± 0.72^bc^	10.05 ± 1.42^cd^	7.70 ± 0.94^c^	46.14 ± 4.28^fg^
**T_9_**	11.84 ± 0.77^b^	10.51 ± 1.01^bc^	8.08 ± 0.91^bc^	59.50 ± 4.80^c^
*Conocarpus lancifolius*				
**T_1_**	0.71 ± 0.13^l^	0.69 ± 0.14^l^	0.33 ± 0.08^m^	5.97 ± 0.99^n^
**T_2_**	0.81 ± 0.14^k^	0.73 ± 0.10^l^	0.39 ± 0.15l^m^	33.67 ± 4.06^j^
**T_3_**	0.87 ± 0.12^kl^	0.80 ± 0.11^l^	0.43 ± 0.14^k^	46.00 ± 5.52^fg^
**T_4_**	5.15 ± 0.23^j^	4.61 ± 0.53^k^	2.99 ± 0.80^j^	6.44 ± 1.11^n^
**T_5_**	5.29 ± 0.59^j^	4.66 ± 0.95^k^	3.62 ± 0.86^hi^	38.00 ± 5.32^i^
**T_6_**	6.10 ± 0.42^hi^	4.96 ± 0.37^jk^	3.83 ± 0.84^hi^	53.22 ± 6.23^d^
**T_7_**	9.17 ± 1.05^f^	7.80 ± 1.47^g^	6.07 ± 0.68^e^	7.22 ± 2.71^mn^
**T_8_**	10.00 ± 0.83^e^	8.70 ± 1.36^f^	6.75 ± 0.51^d^	39.78 ± 3.31^hi^
**T_9_**	10.21 ± 0.80^de^	8.95 ± 1.55^ef^	6.89 ± 0.70^d^	51.89 ± 4.35^de^

DW represents the dry weight of root, shoot, and leaf samples. The values are expressed as the mean ± SD (n = 3). The different letters within a column indicate significant differences between the treatments at p< 0.05.

## 4 Discussion

### 4.1 Total biomass in terms of stem height and diameter, photosynthetic rate, and gaseous parameters

A significant reduction in net photosynthetic rate, stomatal conductance, transpiration rate, and evapotranspiration rate was reported in tested tree species, while a significant elevation was reported in enzymatic and non-enzymatic antioxidant enzymes under different treatment (**Figures 2**–**4** and **Tables 2**, **3**). These results are in accordance with the findings of previous studies as limestone dust and lead accumulation in tree plants significantly reduced growth and development (Inkoom, 2019; Solgi et al., 2020; Antoniadis et al., 2021). For limestone dust, overall photosynthetic reduction, detachment of premature leaves, leaf injuries, and limitation in growth and photosynthetic pigments are the major phenomena reported in various tree species (Inkoom, 2019; Solgi et al., 2020; Szwed et al., 2021). Additionally, the maximum reduction in photosynthetic rate, stomatal conductance, transpiration rate, and evapotranspiration rate was reported in *C. lancifolius* followed by *E. camaldulensis* and *B. ceiba*, which demonstrated that *B. ceiba* and *E. camaldulensis* tree species are more effective bioindicators to limestone dust and Pb toxicity than *C. lancifolius* tree species.

The results of our study demonstrated that total biomass in terms of root and shoot fresh and dry weights, and stem height and stem diameter was reduced with the increase of limestone dust concentration, which might be due to its damaging effects on transpiration, photosynthesis, and respiration (**Figures 2**, **3**). Additionally, the deposit of limestone dust on leaf surface altered the structure of leaves, reduced evapotranspiration, and blocked stomatal pores owing to restricted photosynthetic activities, and overall reduced biomass production (**Figures 2**–**4**). The same results were reported in the previous studies where limestone dust directly affected tree species by causing tissue injuries and restricting photosynthesis and respiration, which can damage foliage production (Midhat et al., 2019; Mohanty and Patra, 2020). Despite the adverse consequences of limestone dust, trees demolished these particulates from the surrounding environment to maintain the air quality. In the main limestone dust-demolishing mechanism, limestone aerosols adhere to tree leaves’ upper and lower parts, while some bounce back when passing through the trees (Heggestad et al., 1972). The bounced particulates deposit elsewhere in soil depending on the size, physical characteristics, deposition, wind velocity, and surface area (Heggestad et al., 1972). Upon interaction with leaves, lime particles with smaller diameters have the tendency to enter directly to the spongy parenchyma of leaf tissues (Sett, 2017), whereas larger-sized lime particles enter the leaf tissues after getting dissolved into carbonic acid when coming into contact with water discharged through leaf stomata and becoming part of tree biomass (Chirenje et al., 2006).

On the other hand, different Pb toxicities in the soil, same as limestone dust toxicities, were reported in the tested species (**Figures 2**–**4**). The plant biomass of *B. ceiba, E. camaldulensis*, and *C. lancifolius* was significantly reduced with the increase of Pb toxicities, which might be due to the inhibited root development and growth under retarding cell division. The same results were reported in previous findings where various levels of Pb damaged plant growth and biomass by inhibiting seed germination, root elongation, seed development, chlorophyll production, transpiration, water potential, osmotic potential, pressure potential, and protein contents (Abdul, 2010; Jiang et al., 2010). Additionally, Pb toxicity sifts the functioning and concentration of several major enzymes of several biochemical reactions like the Calvin cycle of the photosynthesis process, nitrogen metabolic reaction, and sugar metabolic pathways, resulting in a significant effect on the normal functioning of tree plants (Sharma and Dubey, 2005).

### 4.2 Physiological attributes in terms of osmotic stress

Moreover, in our study, the biomass reducing trend in three different tree species under combined toxicities of limestone dust and Pb toxicities was *B. ceiba< E. camaldulensis< C. lancifolius*, demonstrating that *B. ceiba* was more tolerant to both toxicities, while *C. lancifolius* tree species were more sensitive (**Figures 2**–**4**). This might be due to the abundant Pb ions penetrating the cytoplasm of *B. ceiba*; a defensive system is triggered, which protects the cells from the cytotoxicity of the Pb and limestone dust, as reported previously (Breckle and Kahle, 1992). Endocytotic and exocytotic mechanisms respectively mediate these behaviors. The cell’s plasma membrane acts as a “living” barrier, preventing the free inward flow of lead and limestone ions into the cell. Previously, it has been suggested that resuspension of the plasmalemma and certain organelles from dictyosomes and the endoplasmic reticulum (ER) may restrict the free movement of Pb ions in the cytoplasm of *B. ceiba* (Ismail et al., 2013). The vacuole serves as one of the primary storage locations for metals (Sarwar et al., 2017). The presence of cysteine-rich peptides, also known as phytochelatins (PCs), in roots of *B. ceiba* was discovered only after exposure to Pb (Sakthi et al., 2010).

The effect of heavy metals on tree–water relation is well-known for their impacts on water accessibility in the soils, restricted water uptake, root elongation, and other phytotoxic consequences. It has been established that the potential of the root cell sap will be higher than the osmotic potential of soil solution if soils are rich with soluble heavy metal-oriented salts (Upadhyaya et al., 2013). Under these conditions, osmotic stress will be established because water will be unable to move from soil solution to plant roots, which ultimately cause restricted primary root elongation, compromised secondary development, limited root hair surface, and increased root dieback. Our results followed the same conclusion, where the osmotic potential of root cell saps was highest in control plants and decreased with the increase of limestone dust and Pb concentration in air and soil, which emphasized that water was unable to move from soil to plant root and cause osmotic stress in tree plants as depicted in **Figure 4**.

### 4.3 Biochemical attributes in terms of stress enzymes and osmolytes

Heavy metal toxicity in tree species affects a majority of metabolic processes, including the extensive production of reactive oxygen species such as H_2_O_2_ and OH^−1^ (Su et al., 2019). Moreover, heavy metal toxicities cause lipid peroxidation by overproduction of MDA. These ROS and lipid peroxidation trigger oxidative burst to the biomolecules when produced in intensive amounts leading to loss of ions, DNA denaturing and strand damage, protein hydrolysis and denaturing, lipid peroxidation, cell membrane peroxidation, membrane injury and damage, logging in trees, and irreversible metabolic dysfunctioning leading to cell death (He et al., 2017; Wang et al., 2018). To confront this oxidative burst, trees have natural defense mechanisms in the form of antioxidative enzymes, including CAT, SOD, POD, protein, and proline content (Su et al., 2019). SOD is recognized as a pervasive enzymatic antioxidant that plays a vital role in cellular defense mechanisms against ROS. SOD’s major functioning is to restrain the excessive amount of two Haber–Weiss reaction substrates like O_2_ and H_2_O_2_ and minimize the damaging risk of OH radical production, which is recognized as extremely reactive and ultimately cause irreversible damage to cell membranes, DNA, and protein (Wang et al., 2018). In our study, the amount of SOD was increased in all tested tree species by increasing toxic levels of Pb in soils and limestone dust in the air (**Table 3**). This suggests that the toxic effect of heavy metals is possibly exercised through free radical production. The maximum SOD activity was recorded in the combined toxicities of limestone dust and Pb in *B. ceiba* compared to two remaining tree species under the same levels of toxicities, which demonstrated that *B. ceiba* tree species had a strong antioxidant mechanism than *E. camaldulensis* and *C. lancifolius* to detoxify the damaging effects of ROS (**Table 3**). In the same line, *B. ceiba* tree species performed well under heavy metal toxicities by activating antioxidant enzymes and minimizing the oxidative burst more efficiently (Sakthi et al., 2010). Similarly, Sakthi et al. (2010) reported that *B. ceiba* tree species are the most effective heavy metal-tolerant and -absorbent species when grown in an aqueous solution with Pb toxicities. Similarly, CAT antioxidant enzymes are used to alleviate H_2_O_2_ by decomposing it into water and oxygen, but CAT is less effective than POD in detoxifying H_2_O_2_ because of its less substrate affinity to H_2_O_2_. Additionally, CAT is reported to minimize oxidative stress and restore mitochondrial arrangement by improving the potential of mitochondrial membrane, which plays an antiapoptotic role and normalizes replicative and injury healing capability (Guo et al., 2018; Zamponi et al., 2018). Consequently, in less oxidative stress of heavy metals, the tree species mainly activated the antioxidative mechanism by increasing only SOD and POD activities to confront ROS (Siedlecka and Krupa, 2002). In our study, all subjected tree species produced CAT enzymes under the individual as well as the combined toxicities of limestone dust and Pb (**Table 3**). The CAT activity was less in control and increased with the increasing levels of limestone and Pb toxicities, similar to previous studies where CAT activity was enhanced with the increase in heavy metal toxicities (Shen et al., 2017; Steliga and Kluk, 2020). In this work, maximum CAT activity was reported in *B. ceiba* tree species compared to *E. camaldulensis* and *C. lancifolius* under the same levels of toxicities, which emphasized the better handling capacity of *B. ceiba* tree species under Pb and limestone dust toxicities. Additionally, POD enzymes also played a key role in regulating ROS in plants as well as trees (Bela et al., 2015). POD and SOD are antioxidant enzymes but the cell wall of higher tree plants bound their participates in the production of ROS, which is needed for the development and growth of various plant and tree species and has been reported in the cell walls of seedlings, roots, germinated seeds, and leaves but has not been formally detected from the peapods of *B. ceiba, E. camaldulensis*, and *C. lancifolius* (Wang et al., 2018; Su et al., 2019). This emphasized the importance of ROS when produced in required quantities, but overproduction of ROS causes oxidative stress in plants and trees, which are demolished by POD, SOD, and CAT enzymes. Previous studies reported POD enzymes as the main H_2_O_2_-demolishing enzyme after the limitation of APX. POD catalyzed the reduced reaction of H_2_O_2_ and hydroperoxides to alcohols or water molecules to detoxify hazardous organic hydroperoxides (Bela et al., 2015). In our study, POD contents were increased under heavy metal and limestone toxicities in the air and in the soil (**Table 3**). The maximum POD activity was reported in *B. ceiba* followed by *E. camaldulensis* and *C. lancifolius*, which emphasized the effective ROS-scavenging capability of the same species. Similar results were reported in the case of non-enzymatic antioxidants like proline (**Table 2**). The maximum proline contents were reported under limestone dust and Pb toxicities in *B. ceiba* tree species as compared to *E. camaldulensis* and *C. lancifolius*, which emphasized the better phytoremediation capacity of *E. ceiba* trees.

### 4.4 Phytoremediation potential of subjected tree species

Our results showed that the Pb contents of three selected tree species were maximum in roots, shoots, and leaves of *B. ceiba*, followed by *E. camaldulensis* and *C. lancifolius*, respectively. A similar trend was recorded in the case of limestone dust (**Table 4**). The maximum Pb content and limestone dust in *B. ceiba* demonstrated the maximum phytoremediation ability of *B. ceiba*, followed by *E. camaldulensis*. Our findings were supported by the maximum contents of antioxidant enzymes and osmolytes in *B. ceiba* compared to the two remaining tree species, which are indicators of phytoremediation potential of any plant or tree spices (Ur Rahman et al., 2021a). In the same line, previous findings emphasized *Albizia lebbeck* followed by *Millettia peguensis* as a better phytoextractor and phytoremediator as these tree species consumed maximum contents of copper (Cu) and chromium (Cr) and activated the stress enzymes more effectively than the other tree species under different levels of Cr and Cu stresses (Kanwal et al., 2019). Similarly, Mleczek et al. (2017) reported that *Acer platanoides* and *Ulmus laevi* absorbed maximum concentration of Cd, Ag, and Cu compared to the other tree species and performed better compartmentation of these heavy metals in different organs along with efficiently regulating the stress enzymes. Additionally, Yasin et al. (2021) reported that *Morus alba* absorbed maximum contents of Cd, Cu, and Pb in their leaves and bark samples and regulated them more efficiently to minimize their toxic potential in air and soil as compared to *E. camaldulensis*. In conclusion, based on our results, we emphasized that *B. ceiba* tree species are the best phytoextractor and bioindicator for Pb and limestone dust load and must be prioritized to plant in Pb and limestone dust-polluted areas.

## 5 Conclusion

The results of this study showed that the presence of Pb and limestone dust in soils could significantly alter the morphological and physiological traits of *B. ceiba*, *E. camaldulensis*, and *C. lancifolius*. Pb and limestone dust pollution remarkably reduced total biomass, photosynthetic pigments (i.e., chlorophyll a, b, and carotenoids), osmotic potential, water potential, and pressure potential while significantly enhancing the activity of antioxidant enzymes (e.g., CAT, SOD, POD, protein, and proline) through oxidative burst by overproduction of ROS and by producing osmotic stress in root cell saps in all subjected tree species. Although Pb and limestone dust pollution adversely damaged the growth and development of all three tree species, *B. ceiba* proved to be more tolerant than *E. camaldulensis* and *C. lancifolius*. This resulted from the fact that *B. ceiba* could efficiently demolish oxidative stress by activating antioxidant enzymes; minimizing osmotic stress by regulating osmotic, water, and pressure potential; and enhancing photosynthesis by improving transpiration rate, evapotranspiration rate, and stomatal conductance. As efficient phytoextractors, *B. ceiba* and *E. camaldulensis* could be grown in urban areas contaminated by heavy metals and/or limestone dust. Based on the findings of this study, it is recommended that *B. ceiba* and *E. camaldulensis* be utilized as efficient phytoextractors for the remediation of urban areas contaminated by heavy metals and/or limestone dust. Additionally, future research could explore the mechanisms behind the greater tolerance of *B. ceiba* to Pb and limestone dust pollution compared to *E. camaldulensis* and *C. lancifolius*, potentially leading to the development of more resilient plant varieties for use in urban pollution remediation efforts.

## Data availability statement

The original contributions presented in the study are included in the article/supplementary material. Further inquiries can be directed to the corresponding authors.

## Author contributions

SR wrote the original draft and performed the formal analysis. All authors contributed to the article and approved the submitted version.
